# Supplementary figures and images for: Nontargeted and targeted metabolomics approaches reveal the key amino acid alterations involved in multiple myeloma
Source: PeerJ. 2022 Feb 9;10:e12918. doi: 10.7717/peerj.12918 (PMC8840056; doi:10.7717/peerj.12918)

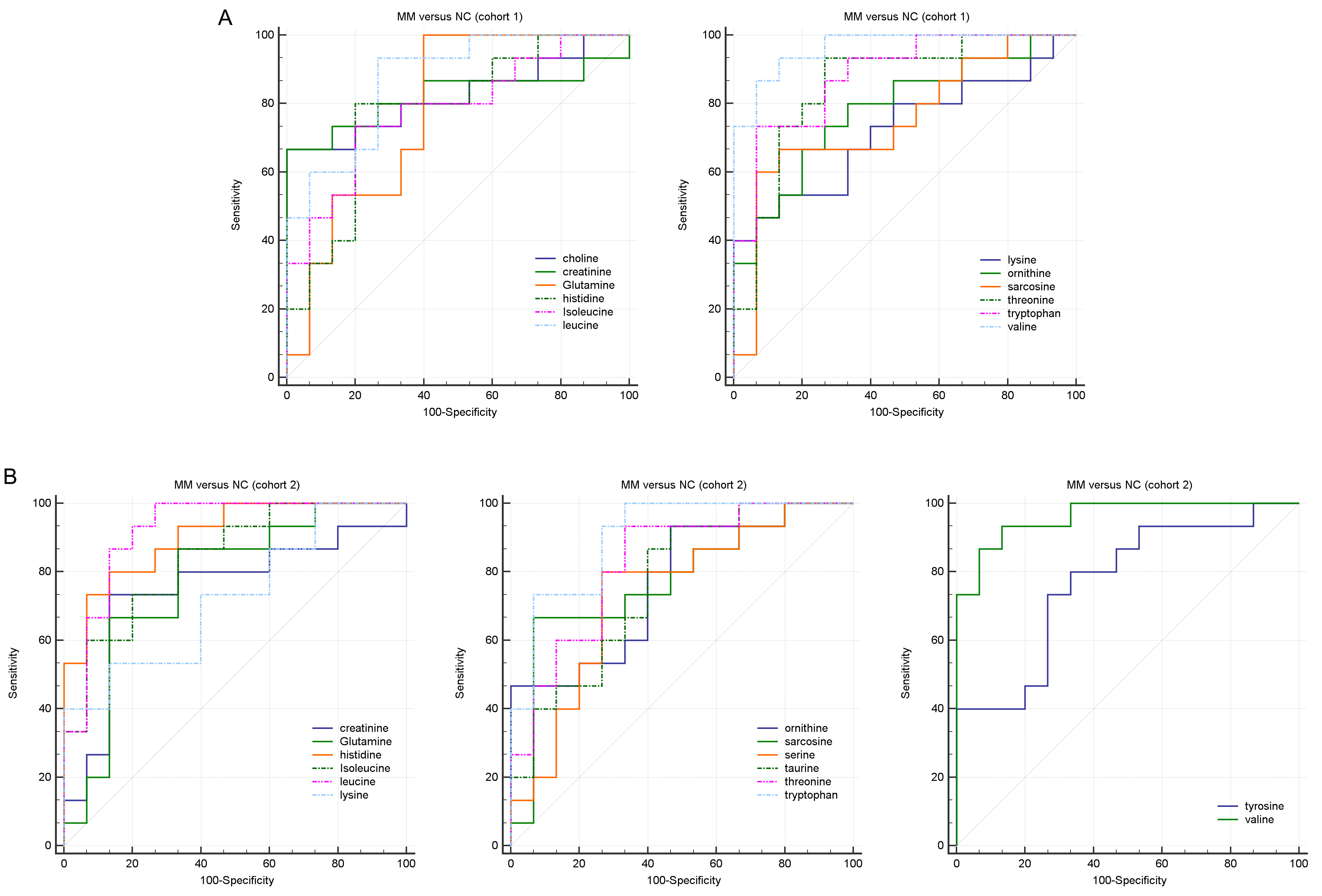

Supplement: Supplemental Information 1 [file peerj-10-12918-s001.jpg]
