# Supplementary material for: Nontargeted and targeted metabolomics approaches reveal the key amino acid alterations involved in multiple myeloma
Source: PeerJ. 2022 Feb 9;10:e12918. doi: 10.7717/peerj.12918 (PMC8840056; doi:10.7717/peerj.12918)
Supplement: Supplemental Information 2 — Abbreviations: ISS, International Staging System. [file peerj-10-12918-s002.docx]

**Supplementary Table 1**. **Clinical characteristics of the 30 participants in cohort 2**

| Group | Age | Sex | ISS |
| --- | --- | --- | --- |
| Normal control (NC, n=15) | 54.36±9.08 | Female (n=6)  Male (n=9) | --- |
| Multiple myeloma (MM, n=15) | 55.60±10.37 | Female (n=5)  Male (n=10) | ISS I (n=2)  1SS II (n=4)  ISS III (n=9) |

Abbreviations: ISS: International Staging System.
